# Perineural dexamethasone as an adjuvant to erector spinae plane block for acute and chronic pain after cardiac surgery

**DOI:** 10.1038/s41598-026-55409-9

**Published:** 2026-06-04

**Authors:** Luis Alberto Rodriguez Linares, Raquel Chacon Ruiz Martinez, Vanessa Henriques Carvalho, Lais Helena Navarro e Lima, Suely Pereira Zeferino, Juliana Oliveira Carneiro, Filomena Regina Barbosa Gomes Galas

**Affiliations:** 1 University of Sao Paulo School of Medicine, São Paulo, Brazil; 2 InCor, São Paulo, Brazil; 3 Hospital Sirio-Libanes, São Paulo, Brazil; 4 IPq-FMUSP, São Paulo, Brazil; 5 UNICAMP, Campinas, Brazil; 6 University of Manitoba, Winnipeg, MB Canada

**Keywords:** Erector spinae plane block, Dexamethasone, Cardiac surgery, Regional anesthesia, Chronic post surgical pain, CPSP, CABG, Cardiology, Diseases, Medical research

## Abstract

Pain control after cardiac surgery remains suboptimal. The erector spinae plane (ESP) block offers a safer regional alternative to neuraxial techniques, but its duration is limited. We tested whether perineural dexamethasone added to bilateral ESP blocks reduces acute postoperative pain intensity and attenuates chronic post surgical pain (CPSP) after coronary artery bypass grafting (CABG). In this randomized, double-blind trial (NCT04313959), 43 patients undergoing elective CABG received bilateral ESP blocks with 0.2% ropivacaine alone (control, *n* = 21) or ropivacaine plus 8 mg perineural dexamethasone (dexamethasone, *n* = 22). Primary outcome: pain intensity at rest on postoperative day 1, measured using the numerical rating scale (NRS). Pre-specified secondary outcomes were mechanical ventilation duration, 48-hour opioid consumption, and pain interference at 30, 60, and 90 days measured using the Brief Pain Inventory (BPI). The primary outcome was not significantly different between groups: NRS pain at rest on day 1 was 1.35 ± 1.95 (control) vs. 1.57 ± 2.27 (dexamethasone), *p* = 0.781 (Fig. 1). Acute pain scores and 48-hour opioid consumption were comparable (Fig. 3). Mechanical ventilation was shorter in the dexamethasone group (median 11.6 vs. 14.7 h; *p* = 0.041; Table 2). At 30 days, no significant between-group differences were found in any BPI domain (all *p* > 0.05; Table 3). At 60 days, pain intensity scores were lower in the dexamethasone group (composite score 0.47 ± 0.88 vs. 1.21 ± 1.28; *p* = 0.019; Table 3; Fig. 2). By 90 days, all outcomes were comparable (all *p* > 0.05). One procedure-related adverse event occurred (pneumothorax, control group). Addition of dexamethasone to ropivacaine did not add to the reduction of acute pain. The secondary exploratory findings of shorter mechanical ventilation time and lower pain intensity at 60 days are hypothesis-generating and should be evaluated using larger, adequately powered multicenter trials with intraoperative blinding and a systemic comparator arm.

Trial registry: NCT04313959.

**Supplementary Information:**

The online version contains supplementary material available at 10.1038/s41598-026-55409-9.

## Introduction

Persistent pain after cardiac surgery is a clinically significant problem. Up to 56% of patients undergoing CABG develop Chronic Post Surgical Pain (CPSP), defined as pain persisting beyond three months not attributable to another cause^1,2^. CPSP impairs functional capacity and reduces quality of life^3^. Effective perioperative analgesia is a key preventive strategy, as uncontrolled acute pain drives central sensitization and increases the risk of chronic pain development^4^.

The ESP block has emerged as a safe and effective regional technique for cardiac surgery, reducing pain and opioid consumption following median sternotomy^5–8^. However, single-injection ESP blocks have limited duration, emphasizing the need for adjuvant strategies. Dexamethasone prolongs peripheral nerve block analgesia through anti-inflammatory and vasoconstrictive mechanisms^9–11^, but its role in fascial plane blocks during cardiac surgery remains undefined^12^. We therefore conducted a randomized, double-blind trial to evaluate whether perineural dexamethasone reduces acute postoperative pain (primary outcome) and whether it shortens mechanical ventilation duration and mitigates CPSP (pre-specified secondary outcomes). The trial followed the CONSORT 2025 guidelines^13,14]^.

## Methods

### Study design and ethical approval

This was a prospective, single-center, randomized, double-blind, placebo-controlled clinical trial conducted at the Instituto do Coração (InCor), University of São Paulo, Brazil. The study was registered on ClinicalTrials.gov (NCT04313959; date of registration: March 18, 2020) and approved by the institutional Research Ethics Committee (protocol No. 3.830.029). All participants provided written informed consent before inclusion.

### Participants

Adults aged > 18 years scheduled for elective coronary artery bypass grafting (CABG) via median sternotomy with left ventricular ejection fraction (LVEF) ≥ 45% were considered for inclusion. Exclusion criteria were allergy to ropivacaine or dexamethasone, coagulopathy, chronic opioid therapy, psychiatric disorders, reoperation, emergency or combined procedures, and refusal to participate. Demographic data, comorbidities, and baseline characteristics were collected from preoperative interviews and medical records.

### Randomization and blinding

Participants were randomly assigned in a 1:1 ratio to receive bilateral erector spinae plane (ESP) blocks with either 0.2% ropivacaine alone (control group) or ropivacaine plus 8 mg perineural dexamethasone (dexamethasone group). Randomization was generated using a computerized sequence and concealed in sequentially numbered, opaque envelopes. The attending anesthesiologist opened the envelope immediately before induction but was not involved in postoperative data collection. Patients, surgeons, intensive care staff, and evaluators remained blinded to group allocation throughout the study.

### Anesthetic management and ESP block technique

General anesthesia was induced with midazolam (0.3–0.35 mg/kg), etomidate (1–2 mg/kg), fentanyl (1–2 mcg/kg), and cisatracurium (0.15 mg/kg), and maintained with sevoflurane (1.5–2%) in 40–50% oxygen/air mixture. No routine prophylactic corticosteroids are administered at our institute for CABG, and no corticosteroids other than the study drug were given to any enrolled patient.

After tracheal intubation and hemodynamic stabilization, bilateral ESP blocks were performed under real-time ultrasound guidance (12–15 MHz linear probe, T5 level) by two experienced regional anesthesiologists each with more than 150 prior ultrasound-guided ESP blocks supervised by the same attending physician throughout. A 22-gauge echogenic needle 100 mm was advanced in-plane to the fascial plane between the transverse process and the erector spinae muscle. Each side received 25 mL of 0.2% ropivacaine (maximum total dose 3 mg/kg). In the dexamethasone group, 4 mg (1 mL) of dexamethasone was added per side (total 8 mg), maintaining the same total injection volume per side. Postoperative ventilation was continued using pressure-controlled or volume-controlled modes per standard ICU protocol without continuous sedative infusions, consistent with our fast-track Enhanced Recovery After Cardiac Surgery (ERACS) pathway.

### Perioperative analgesia

Postoperative analgesia followed an institutional multimodal regimen: metamizole 1 g every 6 h and tramadol 100 mg every 12 h, with intravenous morphine boluses (2–4 mg) available as rescue analgesia. Total opioid and analgesic consumption were recorded for the first 48 h postoperatively. The first patient was enrolled on 20 January 2022.

### Outcome measures

Our primary outcome was acute pain intensity at rest on postoperative day (first morning ICU assessment), measured using the numerical rating scale (NRS).

Our secondary outcomes were: mechanical ventilation duration; 48-hour opioid consumption; all adverse events related to any aspect of the study, including procedure-related complications (pneumothorax, hematoma, vascular puncture, neurologic deficit, or needle-related injury from the ESP block) and drug-related adverse effects; and pain assessed using the Brief Pain Inventory (BPI) at 30, 60, and 90 days postoperatively, evaluating all 7 interference domains (general activity, mood, walking ability, normal work/household activity, relations with others, sleep, and enjoyment of life) and all 4 pain intensity items.

### BPI follow-up methodology

BPI assessments at 30, 60, and 90 days were conducted by a single blinded investigator via structured telephone interview using the validated Brazilian Portuguese version of the BPI. Up to three contact attempts were made at each timepoint before a patient was recorded as missing. At 30 days, one patient per group was unavailable (*n* = 21/group); at 60 and 90 days, all 43 patients were successfully assessed. No data were imputed.

### Sample size calculation

The sample size was calculated based on the primary outcome. Based on data from Krishna et al.^7^ a minimum clinically important difference (MCID) of 1.5 NRS points (SD = 1.5) was used, yielding an effect size (Cohen’s d) of 1.0. With a two-tailed alpha = 0.05 and 80% power, a minimum of 20 patients per group was required; accounting for 5% attrition, 22 patients per group were enrolled. We acknowledge that the assumed effect size was derived from a placebo-controlled comparison and likely overestimates the incremental benefit when comparing two active interventions.

### Statistical analysis

Data were recorded in REDCap and analyzed using IBM SPSS Statistics, version 22.0 (IBM Corp., Armonk, NY, USA). Continuous variables were tested for normality with the Shapiro–Wilk test. Between-group comparisons employed Student’s t-test or Mann–Whitney U test for continuous data and Chi-square or Fisher’s exact test for categorical data. Repeated measures were analyzed with Generalized Estimating Equations (GEE) or Friedman tests, applying Bonferroni correction for multiple comparisons. Between-group BPI comparisons were performed as pairwise Mann–Whitney U tests at each timepoint using individual patient-level raw data. Results are reported as mean ± SD, median [interquartile range], or percentage (%), as appropriate. A two-tailed *p* < 0.05 was considered statistically significant. Given the exploratory nature of all secondary outcomes, findings beyond the primary endpoint are considered hypothesis-generating. Data are available from the corresponding author on reasonable request.

## Results

### Participant flow and baseline characteristics

Forty-four patients were enrolled; one control-group patient was withdrawn before surgery (transfer to palliative care), leaving 43 patients who completed the study (control *n* = 21, dexamethasone *n* = 22). Baseline characteristics were comparable between groups, with a mean age of 64.8 ± 9.0 years in the control group and 63.7 ± 6.6 years in the dexamethasone group (*p* = 0.641). The proportion of male patients was similar between groups (66.7% vs. 81.8%; *p* = 0.280). BMI was slightly higher in the dexamethasone group (27.0 ± 2.6 vs. 25.7 ± 3.2 kg/m^2^; *p* = 0.127). The control group had more patients with non-study drug allergies (23.8% vs. 0%; *p* = 0.021). Other characteristics, including hypertension, diabetes mellitus, and smoking status, were evenly distributed across groups (*p* > 0.5 for all). Full details are presented in Table 1.

**Table 1:** Preoperative baseline characteristics and intraoperative findings.

Variable	Control (*n* = 21)	Dexamethasone (*n* = 22)	*p*-value
Preoperative characteristics			
Age, years (mean ± SD)	64.8 ± 9.0	63.7 ± 6.6	0.641
BMI, kg/m² (mean ± SD)	25.7 ± 3.2	27.0 ± 2.6	0.127
Male patients, n (%)	14 (66.7%)	18 (81.8%)	0.280
Comorbidities			
Hypertension, n (%)	17 (81.0%)	16 (72.7%)	> 0.5
Diabetes mellitus, n (%)	9 (42.9%)	8 (36.4%)	> 0.5
Active smoking, n (%)	11 (52.4%)	11 (50.0%)	> 0.5
Non-study drug allergies^+^, n (%)	5 (23.8%)	0 (0.0%)	0.021*
NYHA class II / III (%)	57% / 43%	59% / 41%	1.000
Intraoperative findings			
Surgical duration, min (mean ± SD)	312 ± 52	312 ± 58	0.992
CPB time, min (mean ± SD)	110.9 ± 30.3	96.2 ± 28.9	0.103
Aortic cross-clamp, min (mean ± SD)	87.1 ± 25.9	76.3 ± 27.0	0.251
Intraoperative fentanyl, mcg (mean ± SD)	618 ± 478	398 ± 329	0.137
Blood glucose at induction, mg/dL	140.5	156.4	NS
Blood glucose after CPB, mg/dL	156.8 ± 45.8	199.5 ± 50.6	0.003*
Blood transfusions, n (%)	4 (19.0%)	2 (9.1%)	0.412

### Intraoperative data

Surgical and anesthetic variables were well balanced between groups (Table 1). Cardiopulmonary bypass (CPB) duration was 96.2 ± 28.9 min in the dexamethasone group and 110.9 ± 30.3 min in the control group (*p* = 0.103). Blood glucose at CPB completion was higher in the dexamethasone group (199.5 ± 50.6 vs. 156.8 ± 45.8 mg/dL; *p* = 0.003).

### Primary outcome: acute pain intensity on postoperative day 1

**Fig. 1:**
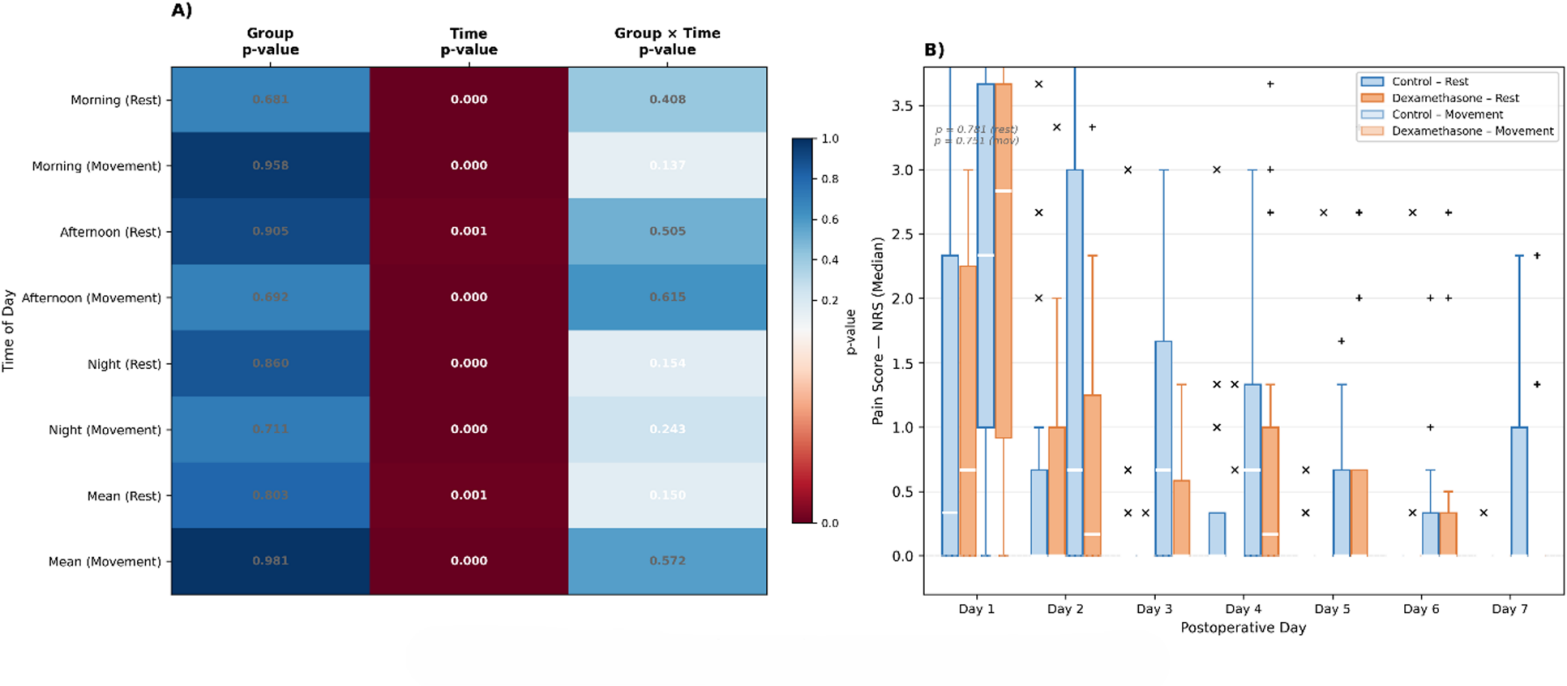
Acute postoperative pain scores (days 1–7). (**A**) GEE model p-values for group, time, and group × time interaction. Group effect was non-significant at all timepoints (*p* = 0.681–0.981). Time effect was significant throughout (*p* ≤ 0.001). Group × time interaction was non-significant (*p* = 0.137–0.615). (**B**) Boxplots of NRS scores at rest and during movement per postoperative day, by group. All between-group comparisons were non-significant (primary outcome: NRS at rest, day 1, *p* = 0.781).

The primary outcome NRS pain score at rest on postoperative day 1 was not significantly different between groups: 1.35 ± 1.95 (control) vs. 1.57 ± 2.27 (dexamethasone), *p* = 0.781 (Fig. 1). GEE analysis confirmed no significant between-group effect across the first seven postoperative days for pain at rest (*p* = 0.781) or during movement (*p* = 0.751), with a significant within-group time effect in both groups (*p* < 0.01), reflecting progressive improvement in pain scores over time.

### Pre-specified exploratory outcomes: postoperative recovery

The dexamethasone group had shorter mechanical ventilation time (mean 751 ± 333 min; median 11.6 h) compared with the control group (848 ± 236 min; median 14.7 h), representing a mean difference of − 97 min (95% CI − 190 to − 5; *p* = 0.041). Both groups exceeded the ERACS-recommended extubation target of 6–8 h post-CABG, and the 97 min difference is of uncertain clinical relevance. ICU length of stay, total hospital stay, drainage volume, and transfusion rates did not differ between groups. Full details are reported in Table 2.

**Table 2:** Key postoperative recovery outcomes.

Variable	Control (*n* = 21)	Dexamethasone (*n* = 22)	Mean diff (95% CI)	*p*
Mechanical ventilation, min (mean ± SD)	848 ± 236	751 ± 333	− 97 (− 190 to − 5)	0.041*
Mechanical ventilation, hours (median)	14.7 h	11.6 h	–	0.041*
ICU length of stay, days (mean ± SD)	3.48 ± 3.33	3.23 ± 1.54	− 0.25 (− 1.7 to 1.2)	0.484
Total hospital stay, days (mean ± SD)	14.8 ± 11.6	11.4 ± 5.5	− 3.4 (− 9.1 to 2.4)	0.435
Ward stay, days (mean ± SD)	11.4 ± 10.6	8.2 ± 4.6	− 3.2 (− 8.3 to 1.9)	0.367
24-h mediastinal drainage, mL	334 ± 261	287 ± 182	− 47 (− 177 to 83)	0.808
Chest drain duration, hours	> 40	> 40	–	0.687

* *p* < 0.05. Both groups exceeded the ERACS extubation target of 6–8 h; the 97-minute difference is of uncertain clinical relevance. ICU = intensive care unit; CI = confidence interval.

### Opioid consumption

Median 48-hour morphine consumption was 0 mg in both groups. Only 2 of 21 control patients (9.5%) and 1 of 22 dexamethasone patients (4.5%) required rescue morphine (*p* = 0.555; Fig. 3). No patient required analgesic interventions beyond the pre-specified multimodal regimen (metamizole, tramadol, and rescue intravenous morphine boluses).

### Long-term pain: BPI at 30, 60, and 90 days

At 30 days, no statistically significant between-group differences were found in any pain intensity item or BPI interference domain (all *p* > 0.05; Table 3). Both groups showed progressive within-group improvement in pain scores over time.

**Fig. 2:**
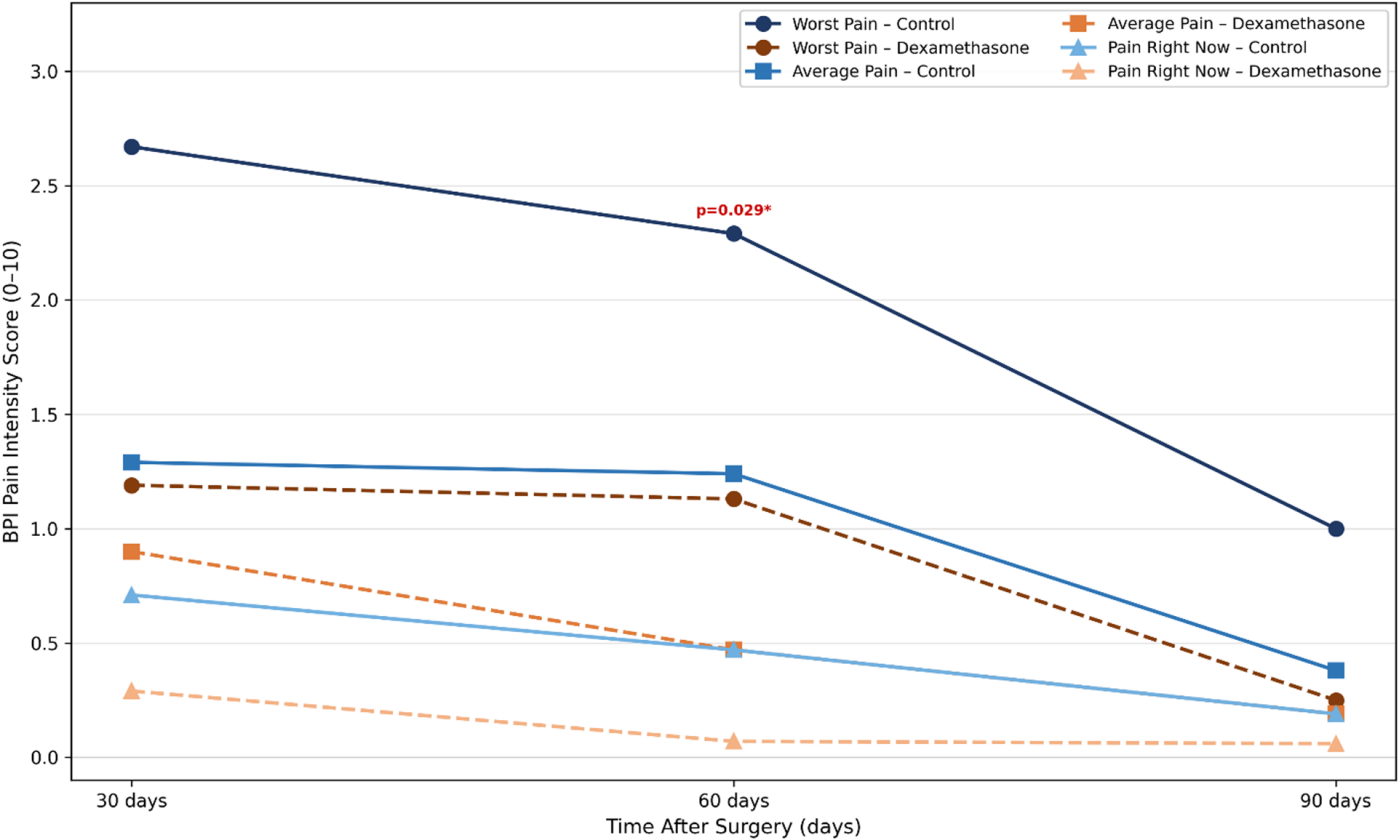
BPI pain intensity scores at 30, 60, and 90 days postoperatively mean BPI pain intensity scores (worst, average, and current pain) at 30, 60, and 90 days by group. Asterisks (*) denote statistically significant between-group differences (Mann-Whitney U, *p* ≤ 0.05): worst pain at 60 days (*p* = 0.029) and weakest pain at 60 days (*p* = 0.010). No significant differences at 30 or 90 days. Solid lines = Control; dashed lines = Dexamethasone.

At 60 days, the dexamethasone group had lower pain intensity scores: worst pain (1.13 ± 2.77 vs. 2.29 ± 2.62; *p* = 0.029), weakest pain (0.20 ± 0.41 vs. 0.82 ± 0.81; *p* = 0.010), and composite pain intensity score (0.47 ± 0.88 vs. 1.21 ± 1.28; *p* = 0.019). No significant between-group differences were found in any of the seven BPI interference domains at 60 days (all *p* > 0.05; Table 3; Fig. 2).

By 90 days, all pain intensity items and BPI interference domains were comparable between groups (all *p* > 0.05; Table 3). More than 90% of patients in both groups reported complete or near-complete pain resolution at this timepoint.

**Table 3:** Brief pain inventory (BPI) all pain intensity items and all 7 interference domains at 30, 60, and 90 days (exploratory secondary outcomes).

BPI domain	30 days			60 days			90 days		
Mean ± SD	Ctrl (*n* = 21)	Dex (*n* = 21)	*P*	Ctrl (*n* = 17)	Dex (*n* = 15)	*p*	Ctrl (*n* = 17)	Dex (*n* = 16)	*p*
Pain intensity scores (0–10 scale)									
Worst pain	2.67 ± 2.73	1.19 ± 1.69	0.069	**2.29 ± 2.62**	**1.13 ± 2.77**	0.029*	1.00 ± 2.16	0.25 ± 0.58	0.358
Weakest pain	1.00 ± 1.00	0.52 ± 0.60	0.130	**0.82 ± 0.81**	**0.20 ± 0.41**	0.010*	0.44 ± 0.63	0.25 ± 0.58	0.292
Average pain	1.29 ± 1.52	0.90 ± 1.09	0.585	1.24 ± 1.44	0.47 ± 0.74	0.062	0.38 ± 0.50	0.19 ± 0.40	0.256
Pain right now	0.71 ± 1.79	0.29 ± 0.72	0.437	0.47 ± 0.80	0.07 ± 0.26	0.055	0.19 ± 0.40	0.06 ± 0.25	0.308
Pain intensity score	1.42 ± 1.57	0.73 ± 0.90	0.181	**1.21 ± 1.28**	**0.47 ± 0.88**	0.019*	0.50 ± 0.81	0.19 ± 0.41	0.244
BPI interference domains (all 7 items, 0–10 scale)									
General activity	1.14 ± 2.33	0.38 ± 1.53	0.068	0.24 ± 0.56	0.00 ± 0.00	0.090	0.18 ± 0.53	0.00 ± 0.00	0.177
Mood	0.76 ± 1.67	0.43 ± 1.54	0.254	0.12 ± 0.49	0.00 ± 0.00	0.363	0.06 ± 0.24	0.00 ± 0.00	0.363
Walking ability	1.43 ± 2.73	0.48 ± 1.54	0.234	0.47 ± 1.12	0.06 ± 0.25	0.294	0.24 ± 0.66	0.00 ± 0.00	0.177
Normal work/household	1.14 ± 2.99	0.62 ± 1.63	0.919	0.35 ± 1.06	0.00 ± 0.00	0.177	0.12 ± 0.49	0.00 ± 0.00	0.363
Relations with others	1.33 ± 2.78	0.43 ± 1.57	0.128	0.35 ± 0.79	0.12 ± 0.50	0.204	0.12 ± 0.33	0.00 ± 0.00	0.177
Sleep quality	1.90 ± 2.81	0.90 ± 1.87	0.134	0.12 ± 0.33	0.12 ± 0.34	0.975	0.18 ± 0.53	0.00 ± 0.00	0.177
Enjoyment of life	0.95 ± 1.96	0.38 ± 1.53	0.071	0.24 ± 0.97	0.00 ± 0.00	0.363	0.00 ± 0.00	0.00 ± 0.00	N/A
Global interference	1.24 ± 2.28	0.52 ± 1.51	0.283	0.27 ± 0.70	0.04 ± 0.10	0.363	0.13 ± 0.30	0.00 ± 0.00	0.090

*Statistically significant between-group comparison (*p* ≤ 0.05), Mann-Whitney U test, two-sided. All p-values from individual patient-level raw data.

BPI = Brief Pain Inventory; Ctrl = control; Dex = dexamethasone. Blinded telephone interview. No data imputed.

Note: p-values at 30 days are between-group (Mann-Whitney U). Previously reported values were within-group Friedman tests and have been corrected.

### Safety and adverse events

One procedure-related adverse event occurred: a pneumothorax in one control-group patient, detected on routine post-operative chest imaging and managed conservatively without chest drain insertion. No other procedure-related adverse events or complications at 90 day follow-up were observed. Two patients (one per group) required transient dialysis for acute kidney injury, both with full recovery. Blood glucose in the dexamethasone group returned to the institutional target range (< 180 mg/dL) within 6 h of ICU admission; no patient required insulin infusion beyond standard protocol, and no glycemic related complications were recorded during the 90-day follow-up.

**Fig. 3:**
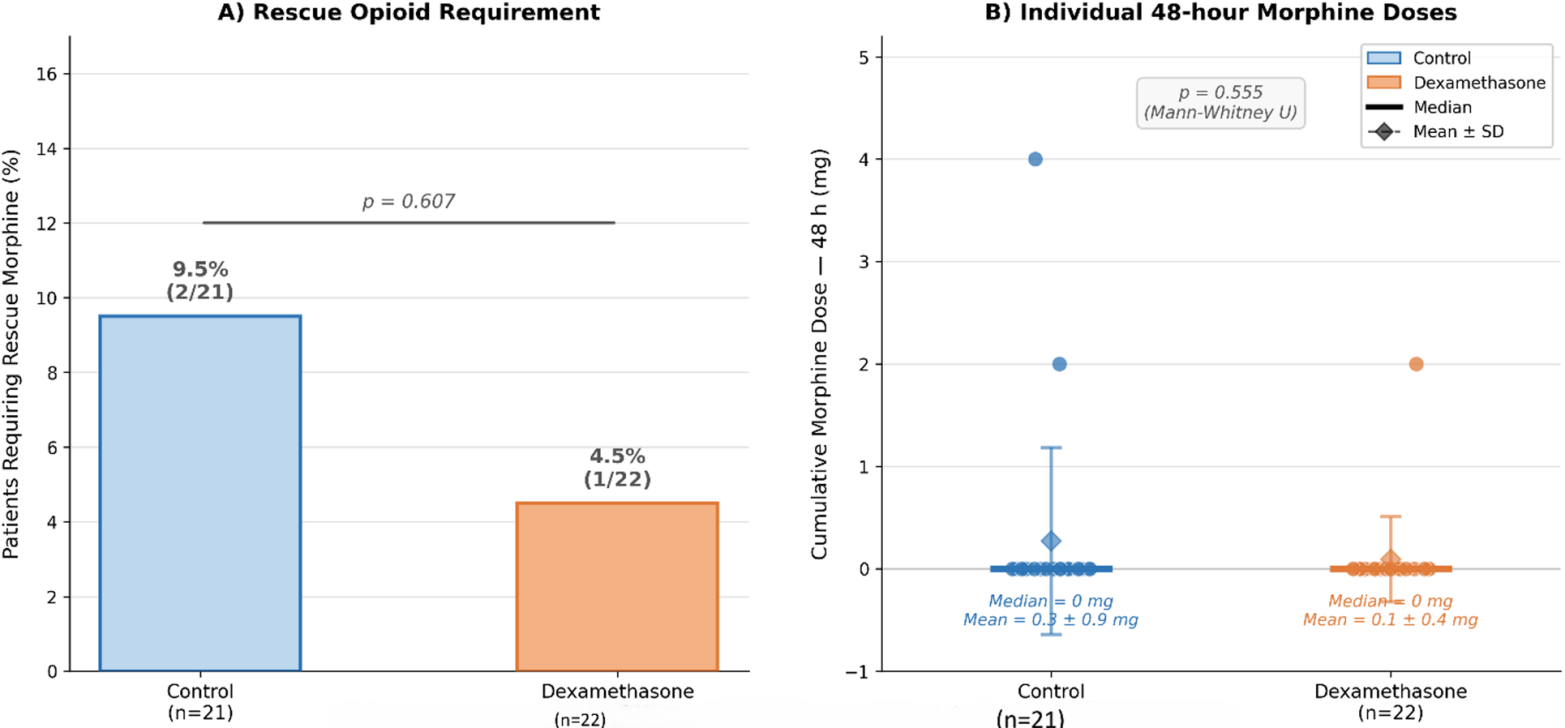
Postoperative opioid consumption (48 h post-extubation). (**A**) Proportion of patients requiring at least one rescue morphine bolus within 48 h of extubation (control 9.5% [2/21] vs. dexamethasone 4.5% [1/22]; *p* = 0.555, Fisher’s exact). (**B**) Individual cumulative 48-hour morphine doses; horizontal bar = group median (0 mg in both groups); diamond = mean ± SD. No statistically significant between-group difference (*p* = 0.555, Mann-Whitney U).

## Discussion

This randomized controlled trial found that adding perineural dexamethasone to bilateral ESP blocks in patients undergoing CABG did not significantly reduce postoperative pain intensity at rest on day 1. Both groups achieved low, comparable pain scores throughout the first postoperative week, and cumulative 48-hour opioid consumption was similarly low, with the median morphine dose being 0 mg in both groups (Figs. 1 and 3).

The absence of a between group difference in acute pain intensity is consistent with the low pain scores observed in both groups, which may have reduced the ability to detect an additive analgesic effect. 15, 16 Furthermore, the assumed effect size (d = 1.0) was derived from a placebo-controlled trial and likely overestimates the incremental benefit when comparing two active interventions. The study was therefore probably underpowered, and the result must be interpreted in this context.

The shorter mechanical ventilation time in the dexamethasone group (median 11.6 vs. 14.7 h; *p* = 0.041; Table 2) represents a pre-specified exploratory finding. Both groups exceeded the ERACS-recommended extubation target of 6–8 h post-CABG^17, 18^, and the 97-minute difference, while statistically significant, is of uncertain clinical relevance. This finding should not be interpreted as confirmatory without replication in a larger trial.

At 60 days, pain intensity scores were lower in the dexamethasone group across three metrics (Table 3; Fig. 2). No corresponding difference was found in any BPI interference domain at this timepoint, and all outcomes converged completely by 90 days. The systemic anti-inflammatory action of absorbed dexamethasone may modulate subacute nociceptive pathways without altering the longer-term mechanisms of CPSP, which are driven by nerve traction, costosternal disruption, and central sensitization.^19^.

The significantly higher blood glucose at CPB completion in the dexamethasone group (199.5 vs. 156.8 mg/dL; *p* = 0.003) is consistent with glucocorticoid pharmacodynamics. In cardiac surgical patients, perioperative hyperglycemia is associated with increased rates of sternal wound infection, new-onset atrial fibrillation, and prolonged ICU stay^20^. Values resolved within 6 h in all patients, and no clinical complications were observed during the 90-day follow-up. Glucose monitoring should therefore be standard when perineural dexamethasone is used in this population.

The absence of a systemic dexamethasone comparator arm prevents attribution of effects to the perineural route specifically. The total dose administered (8 mg) falls within the range known to produce systemic glucocorticoid effects^12^, and systemic absorption from the fascial plane cannot be excluded. Additionally, as blocks were performed under general anesthesia, sensory mapping to confirm dermatomal coverage was not feasible, and inconsistent fascial spread cannot be objectively excluded.

## Strengths and limitations

### Strengths

- Prospective, randomized, double-blind design with validated outcome instruments (BPI) and complete 90-day follow-up.
- Standardized anesthetic and surgical protocols; experienced operators (> 150 prior ESP blocks each) at a consistent thoracic level (T5).
- Complete primary and secondary outcome data without any loss to follow-up.

### Limitations

- The study was likely underpowered: the assumed effect size (d = 1.0) was derived from a placebo-controlled trial and may substantially overestimate the incremental benefit when both arms receive active ESP blocks.
- Intraoperative unblinding of the performing anesthesiologist introduces potential performance bias. Future trials should use pharmacist-prepared identical syringes.
- No sensory mapping was performed under general anesthesia; block adequacy and consistency of fascial spread cannot be objectively confirmed.
- No systemic dexamethasone comparator arm was included; perineural versus systemic contributions cannot be separated.
- Single-center design limits external generalizability.

## Conclusions

Addition of dexamethasone to ropivacaine did not add to the reduction of acute pain in patients undergoing CABG via bilateral ESP blocks. The secondary exploratory findings of shorter mechanical ventilation time and lower pain intensity at 60 days are hypothesis-generating and should be evaluated using larger, adequately powered multicenter trials with intraoperative blinding and a systemic comparator arm.

## Supplementary Information

Below is the link to the electronic supplementary material.

Supplementary Material 1

## Data Availability

Data available from corresponding author on reasonable request.
