# Supplementary material for: Perineural dexamethasone as an adjuvant to erector spinae plane block for acute and chronic pain after cardiac surgery
Source: Sci Rep. 2026 Jun 4;16:25444. doi: 10.1038/s41598-026-55409-9 (PMC13473455; doi:10.1038/s41598-026-55409-9)
Supplement: Supplementary file 1 [file 41598_2026_55409_MOESM1_ESM.pdf]

RESEARCH PROTOCOL

WHO Format

Version 2.0 | Date: January 2026

Version 2.0 (January 2026): Protocol updated to align with the revised manuscript following peer review. Nine corrections were made; a full change log is provided at the end of this document. The original protocol (Version 1.0, December 2025) is retained on file.

1. General Information

**Protocol title**	Dexamethasone as an Adjuvant to Erector Spinae Plane Block in Cardiac Surgery: A Randomized Controlled Trial on Postoperative Recovery and Chronic Post Surgical Pain
**Clinical trial registration**	ClinicalTrials.gov, ID: NCT04313959. Registration date: 16 March 2020. Study start (first patient enrolled): 20 January 2022.
**Protocol version and date**	Version 2.0 — January 2026 (updated following peer review to align with revised manuscript). Version 1.0 — December 2025 (original submission protocol, retained on file).
**Principal Investigator**	Dr. Luis Alberto Rodriguez Linares, MD, PhD. University of Sao Paulo School of Medicine / Instituto do Coracao (InCor), Sao Paulo, Brazil. E-mail: luisro0729@hotmail.com
**Co-Investigators**	Raquel Chacon Ruiz Martinez, Vanessa Henriques Carvalho, Lais Helena Navarro e Lima, Suely Pereira Zeferino, Juliana Oliveira Carneiro, Filomena Regina Barbosa Gomes Galas.
**Study site**	Instituto do Coracao (InCor), University of Sao Paulo, Sao Paulo, Brazil.
**Sponsor and funding**	Self-funded by the investigators. No external commercial or governmental funding.
**Ethics approval**	Approved by the institutional Research Ethics Committee, protocol No. 3.830.029.

2. Background and Rationale

Median sternotomy for CABG is associated with substantial acute chest wall pain and a high incidence of Chronic Post Surgical Pain (CPSP) — defined as pain persisting beyond three months after surgery not attributable to another cause (Pogatzki-Zahn et al., IASP, 2025). Up to 56% of patients undergoing CABG develop CPSP, which impairs recovery, limits functional capacity, and reduces quality of life.

Thoracic epidural analgesia can improve pain control but is limited in cardiac surgery due to anticoagulation and the risk of neuraxial hematoma. The erector spinae plane (ESP) block has emerged as a safer regional alternative, reducing pain and opioid consumption and potentially facilitating early extubation, but its duration remains limited with single-injection techniques.

Dexamethasone is a well-established adjuvant that prolongs peripheral nerve block analgesia through anti-inflammatory and vasoconstrictive mechanisms. However, its role in fascial plane blocks during cardiac surgery remains undefined. There is a clear knowledge gap regarding whether perineural dexamethasone added to ESP blocks can enhance perioperative recovery and reduce the risk of CPSP after CABG.

3. Objectives

Primary Objective

To determine whether adding perineural dexamethasone (8 mg total) to bilateral ESP blocks reduces postoperative pain intensity, defined as the NRS pain score at rest on postoperative day 1 (first morning ICU assessment), in patients undergoing elective CABG via median sternotomy.

Secondary Objectives

- To evaluate the effect of perineural dexamethasone on the duration of mechanical ventilation.
- To compare 48-hour postoperative opioid (morphine) consumption between groups.
- To assess subacute and chronic pain at 30, 60, and 90 days using the Brief Pain Inventory (BPI), evaluating all four pain intensity items and all seven functional interference domains.
- To describe and compare all adverse events related to any aspect of the study, including procedure-related complications (pneumothorax, hematoma, vascular puncture, neurologic deficit, needle injury from ESP block) and drug-related adverse effects (hyperglycemia, infection, wound complications).
- To compare ICU and hospital length of stay between groups.

4. Study Design

Prospective, single-centre, randomized, double-blind, controlled clinical trial with two parallel groups (1:1 allocation ratio):

- Control group: ESP block with 0.2% ropivacaine alone.
- Dexamethasone group: ESP block with 0.2% ropivacaine plus 8 mg perineural dexamethasone (4 mg per side).

The trial was conducted and reported in accordance with the CONSORT 2025 guidelines for randomized studies (Butcher et al., BMJ, 2025).

5. Study Population

Target Population

Adult patients undergoing elective CABG via median sternotomy with preserved left ventricular function at Instituto do Coracao (InCor), University of Sao Paulo.

Inclusion Criteria

- Age >18 years.
- Scheduled for elective CABG via median sternotomy.
- Left ventricular ejection fraction (LVEF) >=45%.
- Ability to provide written informed consent.

Exclusion Criteria

- Allergy to ropivacaine or dexamethasone (study drugs).
- Coagulopathy or anticoagulation precluding regional technique.
- Chronic opioid therapy (pre-existing opioid dependence).
- Relevant psychiatric disorders.
- Reoperation, emergency surgery, or combined procedures.
- Refusal to participate or inability to provide consent.

6. Interventions

ESP Block Technique (Both Groups)

- Timing: after induction of general anesthesia and hemodynamic stabilization.
- Ultrasound-guided bilateral ESP block at T5 level using a 12-15 MHz linear probe.
- Needle: 22-gauge echogenic needle advanced in-plane to the fascial plane between the erector spinae muscle and the transverse process.
- Injection: 25 mL per side of 0.2% ropivacaine (maximum total dose 3 mg/kg).
- Dexamethasone group only: 4 mg (1 mL) dexamethasone added per side (total 8 mg), maintaining the same overall injection volume of 26 mL per side.
- Control group: 0.2% ropivacaine only, same volume and technique.
- Operators: two experienced regional anesthesiologists, each with >150 prior ultrasound-guided ESP blocks, supervised by the same attending physician throughout the study.

Perioperative Management

- Standardized general anesthesia: midazolam (0.3-0.35 mg/kg), etomidate (1-2 mg/kg), fentanyl (1-2 mcg/kg), cisatracurium (0.15 mg/kg), maintenance with sevoflurane (1.5-2%) in 40-50% oxygen/air.
- Standard CPB management and surgical technique performed by the same cardiac surgical team.
- Corticosteroids: routine prophylactic corticosteroids are not part of the standard CABG protocol at InCor; no corticosteroids beyond the study drug were administered to any patient.
- Postoperative ventilation: pressure-controlled or volume-controlled mechanical ventilation in the ICU without continuous sedative infusions, consistent with the fast-track ERACS pathway. Short-acting induction agents had dissipated prior to ICU arrival.
- Standardized multimodal postoperative analgesia: metamizole 1 g every 6 hours and tramadol 100 mg every 12 hours, with intravenous morphine boluses (2-4 mg) as rescue analgesia.

7. Outcomes

Primary Outcome

NRS pain score at rest on postoperative day 1, defined as the first morning assessment performed in the ICU — a single outcome variable measured at a single pre-specified time point. This is the primary hypothesis-testing endpoint of the trial.

Pre-specified Secondary Outcomes

- Duration of mechanical ventilation (minutes from ICU admission to tracheal extubation).
- Cumulative opioid (morphine equivalent, mg) consumption over 48 hours post-extubation.
- All adverse events related to any aspect of the study, including: procedure-related complications (pneumothorax, periprocedural hematoma, vascular puncture, neurologic deficit, needle-related injury from the ESP block) and drug-related adverse effects (hyperglycemia, surgical site infection, wound-healing complications, corticosteroid-related sequelae).
- BPI scores (all four pain intensity items and all seven interference domains: general activity, mood, walking ability, normal work/household activity, relations with others, sleep, and enjoyment of life) at 30, 60, and 90 days postoperatively.
- ICU and total hospital length of stay.

Exploratory Measures

Additional NRS assessments (during movement, days 2-7) are collected as exploratory data and are not part of hypothesis-testing analyses.

8. Sample Size

The sample size was calculated based on the primary outcome: NRS pain score at rest on postoperative day 1. Based on data from Krishna et al. (J Cardiothorac Vasc Anesth, 2019), a minimum clinically important difference (MCID) of 1.5 NRS points (SD = 1.5) was considered clinically relevant, yielding an effect size (Cohen's d) of 1.0. With a two-tailed alpha = 0.05 and 80% power, at least 20 patients per group were required. Allowing for approximately 5% attrition, 22 patients per group were planned (n=44 total). Forty-three patients completed follow-up (21 control, 22 dexamethasone; one patient in the control group was withdrawn before surgery due to transfer to palliative care).

Limitation acknowledged: the effect size assumption (d = 1.0) was derived from a placebo-controlled ESP trial and may overestimate the incremental benefit when both groups receive active ESP blocks. This trial should therefore be considered a pilot study; the results will inform the power calculation for a future adequately powered multicenter trial.

9. Randomization and Blinding

Randomization was performed using a computer-generated sequence with 1:1 allocation, concealed in sequentially numbered, opaque, sealed envelopes.

Blinding was maintained as follows:

- Patients, surgeons, ICU nursing staff, and all outcome assessors were blinded to group allocation throughout the study.
- The anesthesiologist performing the ESP block opened the allocation envelope immediately before the procedure and was therefore aware of group allocation during surgery. This anesthesiologist was not involved in any postoperative data collection or outcome assessment. This represents a recognized limitation (potential performance bias in intraoperative opioid administration) and is acknowledged in the manuscript.
- Future trials should use pharmacist-prepared, identical-appearance syringes to maintain intraoperative blinding.

Note: the original Version 1.0 protocol stated that 'study syringes were prepared identically to maintain blinding.' This was inaccurate as written; the intraoperative anesthesiologist was not blinded after envelope opening. This Version 2.0 corrects that statement for accuracy.

10. Study Procedures and Follow-up

Screening and Enrolment

- Eligibility confirmed during preoperative assessment, at least 24 hours before surgery.
- Written informed consent obtained before any study procedure.

Baseline Data Collection

- Demographics, comorbidities, NYHA functional class, preoperative medications, and baseline pain assessment.
- Laboratory data: creatinine, urea, electrolytes, hemoglobin, platelet count, INR.

Perioperative Data (Intraoperative)

- Anesthetic agents and doses, CPB duration, aortic cross-clamp time, intraoperative opioid consumption, blood glucose at induction and at CPB completion, intraoperative complications.

Postoperative Data (In-hospital, Days 0-7)

- NRS pain scores at rest and during movement (exploratory): ICU arrival, 2h, 4h, 6h, 8h, 12h, 24h; then morning, afternoon, and evening on days 1-7 or until discharge, whichever comes first.
- Primary outcome assessment: NRS at rest on the morning of postoperative day 1 (first morning ICU assessment), performed by the blinded outcome assessor.
- Opioid and analgesic consumption: daily for 48 hours post-extubation.
- Mechanical ventilation time, ICU and hospital length of stay.
- Adverse events, complications, and safety data.

Long-term Follow-up: BPI at 30, 60, and 90 Days

BPI assessments are conducted by a single blinded outcome assessor (not a member of the treating team) using the validated Brazilian Portuguese version of the Brief Pain Inventory. Assessors work from a separate patient list without access to group allocation to maintain blinding.

- Mode of collection: structured telephone interview following a standardized script.
- Contact protocol: up to three contact attempts at each time point. If a patient is unreachable after three attempts, they are classified as missing for that time point; data are not imputed.
- All seven BPI interference domains (general activity, mood, walking ability, normal work/household activity, relations with others, sleep, enjoyment of life) and all four pain intensity items (worst, weakest, average, current pain) are assessed at each time point.
- Missing data: reported transparently; no imputation performed.

11. Data Management and Statistical Analysis

Data are recorded in REDCap (Research Electronic Data Capture) and exported for analysis in IBM SPSS Statistics version 22.0 (IBM Corp., Armonk, NY).

- Continuous variables: normality assessed via Shapiro-Wilk test; compared by Student's t-test or Mann-Whitney U test as appropriate.
- Categorical variables: Chi-square test or Fisher's exact test.
- Primary outcome: between-group comparison of NRS at rest on day 1 using the Mann-Whitney U test (two-tailed, alpha = 0.05).
- Repeated measures pain scores (NRS days 1-7): analyzed with Generalized Estimating Equations (GEE) to account for repeated assessments and test group-by-time interaction.
- BPI outcomes (30, 60, 90 days): between-group comparisons performed as pairwise Mann-Whitney U tests at each individual time point using individual patient-level raw data. Within-group longitudinal change analyzed with the Friedman test.
- Significance level: two-tailed $p < 0.05$ for the primary outcome. All secondary and BPI comparisons are exploratory; no correction for multiplicity is applied, and findings are interpreted as hypothesis-generating.
- Missing data: described but not imputed. Sensitivity analyses are not pre-specified given the small sample size.
- Given the small sample size, formal multivariable regression adjustment is not performed; future larger trials should incorporate pre-specified covariate adjustment for BMI and other imbalanced variables.

12. Ethical Considerations

The study was conducted in compliance with the Declaration of Helsinki and applicable local regulations. Ethics approval was obtained from the institutional Research Ethics Committee (protocol No. 3.830.029) prior to enrolment of the first patient.

- Risks: ESP block-related complications (pneumothorax, hematoma, vascular puncture, neurologic deficit — all rare); transient glucocorticoid-related hyperglycemia; standard surgical and anesthetic risks.
- Benefits: potential for improved analgesia and faster recovery; contribution to evidence on the use of ESP blocks with adjuvants in cardiac surgery.
- Informed consent: obtained before any study procedure, with full explanation of the nature of the study, procedures, risks, benefits, and the right to withdraw at any time without prejudice to clinical care.

- Confidentiality: participant data are de-identified and stored securely; access is restricted to the study team.
- Safety monitoring: adverse events are recorded continuously and reviewed by the principal investigator. There was no formal Data Safety Monitoring Board for this pilot study; a DSMB will be incorporated in future adequately powered trials.

13. Dissemination and Data Sharing

Results will be submitted for publication in peer-reviewed journals and presented at national and international scientific meetings. Reporting will follow the CONSORT 2025 guidelines.

Data availability: individual patient-level data are available from the corresponding author (luisro0729@hotmail.com) on reasonable written request, subject to ethics committee approval and a data sharing agreement.

Appendix: Change Log — Version 1.0 to Version 2.0

The following table documents all changes made in this revision. Changes were made to align the protocol with the revised manuscript following peer review.

#	Section	Issue in Version 1.0	Correction in Version 2.0
1	Title	Critical: 'Pain Chronification' used — not the IASP definition	Updated to 'Chronic Post Surgical Pain (CPSP)' per IASP 2025 terminology
2	Sec. 1	Critical: Registration date left as '[insert date]' — blank field	Registration date filled: 16 March 2020 (NCT04313959)
3	Sec. 1	Critical: Version 1.0 dated Dec 2025 while study started Jan 2022 — inconsistent	Protocol updated to Version 2.0; Version 1.0 retained on file with explanation
4	Sec. 3 & 7	Critical: Primary outcome defined as VAS/NRS at rest and movement across 7 days (14 measurements) — multiple items and timepoints	Primary outcome redefined as NRS at rest on postoperative day 1 (single item, single timepoint)
5	Sec. 9	Critical: States 'syringes prepared identically to maintain blinding' — inaccurate; block anesthesiologist was not blinded intraoperatively	Corrected: anesthesiologist opened envelope before procedure and was not blinded; this is acknowledged as a limitation; future trial recommendation added
6	Sec. 3	Minor: Typo 'Objetives' instead of 'Objectives'	Corrected to 'Objectives'
7	Sec. 2 & 6	Moderate: 'Pain chronification' and 'chronic post-sternotomy pain' used without CPSP terminology; corticosteroid and	CPSP terminology updated throughout; InCor corticosteroid protocol clarified; postop ventilation without sedative infusions explained

		ventilation protocols not specified	
8	Sec. 10	Moderate: BPI follow-up described as 'telephone or outpatient assessment' without protocol detail (mode, blinding, contact attempts, missing data)	Detailed BPI follow-up subsection added: telephone interview, blinded assessor, 3 contact attempts, missing data not imputed, all 7 domains assessed
9	Sec. 7	Moderate: Adverse events defined as 'related to ESP block or dexamethasone' — too narrow; excludes needle injury and other procedure complications	Adverse events broadened to include all study-related complications including needle injury from ESP block

---

End of Research Protocol — Version 2.0
